# Socioeconomic Characteristics and Trends in the Consumption of Ultra-Processed Foods in Korea from 2010 to 2018

**DOI:** 10.3390/nu13041120

**Published:** 2021-03-29

**Authors:** Jee-Seon Shim, Sun-Young Shim, Hee-Jeung Cha, Jinhee Kim, Hyeon Chang Kim

**Affiliations:** 1 Department of Preventive Medicine, Yonsei University College of Medicine, Seoul 03722, Korea; CHJ7114@yuhs.ac (H.-J.C.); hckim@yuhs.ac (H.C.K.); 2 Department of Public Health, Graduate School, Yonsei University, Seoul 03722, Korea; ssy951004@yuhs.ac; 3 Department of Preventive Medicine and Public Health, Ajou University School of Medicine, Suwon 16499, Korea; jhkim06@ajou.ac.kr

**Keywords:** ultra-processed foods, trends, socioeconomic characteristics, Korean, 24-h recall, NOVA food classification

## Abstract

There is growing evidence for a global transition to a more highly processed diet. While the dietary share of ultra-processed foods depends on a country’s economic status, food choice and consumption are also influenced by the socioeconomic situation of individuals. This study investigated whether ultra-processed food consumption differed across socioeconomic subgroups and over time (2010–2018) in Korea. Cross-sectional data from the Korea National Health and Nutrition Examination Survey 2010–2018 were analyzed. Food and beverages reported in a one-day 24 h recall were classified according to the NOVA food classification criteria. The dietary energy contribution of ultra-processed foods was high among men and urban residents, and increased with education and income level; additionally, it reached its peak in adolescents and thereafter decreased with increasing age. After adjusting the socioeconomic variables, such associations remained significant, except for income level. The overall contribution of ultra-processed foods increased from 23.1% (2010–2012) to 26.1% (2016–2018), and the same trend over time was observed in all age groups and socioeconomic strata. In the Korean population, ultra-processed food consumption differed by individual socioeconomic characteristics, but gradually increased over time, and this trend was consistently found in all socioeconomic subgroups. Future strategies to promote healthy food choices are needed for the Korean population.

## 1. Introduction

Ultra-processed foods are products manufactured industrially with substances extracted or refined from natural foods and chemical additives [1]. These foods include breakfast cereals, sugared drinks, confectionary, meat and fish products (e.g., chicken nuggets), and various types of convenience foods. Ultra-processed foods have poor nutrient profiles, such as having high energy density, being high in sugar, sodium, and fat, and being low in protein, fiber, minerals, and vitamins [2,3,4]. They are also cheap, highly palatable, conveniently consumed with minimal (or no) preparation anywhere and anytime, and having less satiety due to their own physical characteristics, often leading to overconsumption [5,6,7,8]. Additionally, the consumption of ultra-processed foods is associated with low dietary quality, as well as unhealthy lifestyle-related behavior such as physical inactivity and smoking [4,9,10,11]. Recently, there has been growing evidence of adverse health impacts of the consumption of ultra-processed foods [9,12,13,14,15].

The dietary energy contribution of ultra-processed foods has been reported to be generally high in high-income countries such as the United States (58.8%) [16], the United Kingdom (56.8%) [17], and Canada (47.7%) [3], and relatively low in relatively low-income countries such as Brazil (20.4%) [4], Mexico (29.8%) [18], and Columbia (15.9%) [19]. However, ultra-processed food product sales worldwide are on an upward trend [20,21], and ultra-processed food purchases also have a noticeable increasing tendency in several household food expenditure surveys [22,23,24]. Changes in demographic and sociocultural characteristics (i.e., increased income, urbanization, changing labor market, and an increase in the number of women working outside the home) that are brought along with economic growth basically drive the demand for ultra-processed foods [21,25]. With improvements in innovative food technologies for processing and packaging, more diverse types of ultra-processed foods have been produced [26]. All of the supply chains involved in the production of raw foods, manufacturing, marketing, and consumption are controlled by a small number of transnational food and beverage manufacturers and by a strong power of transnational grocery retailers and fast-food companies, resulting in such market power which is potentially instigating a global transition to ultra-processed food [21]. In addition, although a policy intervention to minimize the negative impact of an unhealthy diet is needed, political and regulatory actions for these supply-side sectors of the food system are still weak [21]. Ultra-processed foods seem to penetrate deeply into society and to rapidly replace freshly prepared meals [20,22,24,27,28,29]. While socioeconomic status is known to be a major determinant of the consumption of these foods, human diet is influenced by many other factors other (e.g., culture) and thus, the vulnerable groups (i.e., those more likely to consume ultra-processed foods) differ from country to country [3,9,16,30,31,32].

Thus far, little is known about ultra-processed food consumption in the Korean population. According to one of the few recently conducted studies by the authors of the current study [33], the proportion of ultra-processed foods in the diet of Korean adults accounts for 25.1% of total energy intake. High consumption of ultra-processed foods is associated with poor nutrient intake, as well as poor dietary quality. Along with economic growth, like other countries, the Korean diet has changed consistently over time, but still has several unique characteristics (i.e., relatively high vegetable intake and low fat intake) [34,35]. The sales of the ultra-processed foods and beverages of Korea are outstandingly low among high-income countries [21], and the mean consumption of sugar-sweetened beverages, a major type of ultra-processed food, has been reported to be relatively low compared to other high-income countries [36]. However, Korea is still under the influence of a global transition.

Thus, this study aimed to evaluate whether the ultra-processed food consumption of Koreans differed in terms of socioeconomic characteristics and whether there were time trends in ultra-processed food consumption from 2010 to 2018 in Korea.

## 2. Materials and Methods

### 2.1. Data Source and Study Population

We used data from three cycles of the Korea National Health and Nutrition Examination Survey (KNHANES): 2010–2012, 2013–2015, and 2016–2018. KNHANES is a continuous, nationwide, cross-sectional survey to assess the health and nutritional status of Koreans. KNHANES comprises health interviews, health examinations, and nutrition surveys. KNHANES sampling follows a multistage stratified clustered probability design. Each year, 192 primary sampling units were selected from about 200,000 small geographical areas covering the whole country. Using systematic sampling, 20–23 target households were sampled for each primary sampling unit consisting of 60 households. Within the selected households, all individuals aged one year or older were invited to KNHNAES. Thus, in each of the studied years, approximately 10,000 non-institutionalized Koreans aged one year or older were recruited as representative samples. The participation rate was approximately 75% and more details are available elsewhere [37,38].

The nutrition survey was carried out approximately one week after the health interviews and health examination surveys. Trained dietitians visited the participants’ homes and collected information concerning dietary habits and intakes in an in-person interview. Dietary intake was assessed by a one-day 24 h recall. During the recall, the respondents were requested to report details on the foods and beverages they consumed over the previous 24 h (e.g., food description, quantity, and time and place of eating). The quantity of the food and beverages consumed was required to be reported in terms of volume on the basis of various measuring tools (e.g., standard measuring cups, spoons, a ruler, and two-dimensional drawings of measuring guides). If single food items were consumed, more specific information on the food (e.g., food status and brand name) with generic food names were collected. In the case of homemade dishes, the unique home recipe was collected from the person in charge of cooking. The multiple-pass method designed for complete and accurate collection of dietary data such as each respondent’s food consumption and home recipes was applied throughout the interview. For children or those who had difficulty in reporting their diet, the interview was completed with the help of others (e.g., mother or caregiver). The dietary data from the completed one-day 24 h recall were coded and edited using the software developed for the KNHANES. After data entry, multi-ingredient foods through culinary preparation were disaggregated into their raw ingredients. In this process, for homemade foods, each household’s unique recipe collected from the person in charge of cooking was applied and, for foods prepared at school or work cafeterias or restaurants, the standard recipes developed for the data processing of the KNHANES were applied. The amount of food consumed was converted into weight (g) using the database for volume and weight of foods [38,39]. The dietary energy and nutrient intake from each food item was calculated using Korean food composition data, updated regularly by the National Institute of Agricultural Sciences and the Korea Centers for Disease Control and Prevention (KCDC) [40,41]. All survey protocols and procedures were approved by the Institutional Review Board (IRB) of the KCDC and informed consent was obtained from each participant before the survey. The raw data file and documentation of this survey were released by KCDC (https://knhanes.cdc.go.kr, accessed on 20 February 2020). More details of the rationale, design, and methods have been described elsewhere [37,38,39,41].

A total of 72,751 individuals participated in at least one among three component surveys of KNHANES from 2010 to 2018. We excluded those who did not participate in a one-day 24 h recall (*n* = 7878), pregnant and breastfeeding women (*n* = 768), and those with missing information, including education and household income (*n* = 6682). Thus, 57,423 individuals aged one year or older were analyzed in this study (*n* = 20,461 in 2010–2012; *n* = 17,746 in 2013–2015; *n* = 19,216 in 2016–2018).

### 2.2. Food Classification According to the NOVA Classification Criteria

All reported food and beverage items were classified according to NOVA, the most widely used food classification method [1,8]. This classification system categorizes foods into four groups on a basis of the nature, extent, and purpose of industrial food processing: Unprocessed or minimally processed foods (*n* = 10 food subgroups); processed culinary ingredients (*n* = five food subgroups); processed food (*n* = seven food subgroups); and ultra-processed foods (*n* = 12 food subgroups).

In brief, NOVA group 1 includes original foods obtained from nature (unprocessed) or natural food after minimal processing, such as removing inedible parts, washing, chilling, refrigeration, freezing, crushing, grinding, filtering, drying, roasting, boiling, placing in containers, or vacuum-packaging. NOVA group 2 is of processed culinary ingredients used in the seasoning of raw foods (e.g., salt, sugar, honey, oils, and fats). They are usually obtained from group 1 foods through industrial processes such as extracting, mining, pressing, or centrifuging. NOVA group 3 comprises processed foods that are made by adding group 2 foods to group 1 foods, aiming to preserve natural foods longer or to enhance their palatability. They include salted, sugared, pickled, canned, or bottled foods, and processed cheese. Such processed products are recognized as new versions of original foods because they retain most of the basic characteristics of the original foods. Finally, NOVA group 4 consists of ultra-processed foods of a main concern in this study. They include breakfast cereals, breads and cakes, cookies, sweet or salty snacks, candies, chocolates, desserts, ice cream, sugary milk and fruit and vegetable drinks, soft drinks, sugared teas and coffee, distilled alcoholic beverages, meat and fish products (e.g., chicken nuggets), instant sauces and spreads, sweeteners, instant formulas and baby products, and diverse sorts of ready-to-eat or ready-to-heat foods (e.g., instant rice, noodles, soups, dumplings, or pizza). These foods are durable, hyper-palatable, and highly profitable food products that are manufactured mostly (or entirely) of cheap industrial substances extracted or derived from foods and additives through a highly complicated process, with little or no whole food content. The rationale and details on each NOVA food group have been described elsewhere [1,8].

In the data of KNHANES from 2010 to 2018, a total of 4927 food items were reported to be consumed as food itself or as ingredients. Three investigators classified each food item according to the NOVA classification criteria, and for some items with discrepant classification, they were resolved by discussion. Thus, all food items were mutually exclusively categorized into one of the four NOVA food groups and the subgroups within each of these NOVA groups.

### 2.3. Sociodemographic Variables

This study included sex, age, residence area, education status, and income as sociodemographic variables. Such information was provided by adult respondents aged 19 years or older from a sample household during the health interview survey. For income, to take into account the difference in a household size, we utilized the equivalized household income, calculated by dividing the annual household income by the square root of the number of household members. The sociodemographic variables were categorized as follows: Sex (male and female), age (1–12 years, 13–19 years, 20–49 years, 50–64 years, and 65 years or older), residence area (urban and rural), education status (middle school or less, high school, and college or higher), and income (low: quartile 1, middle: quartile 2–3, and high: quartile 4).

### 2.4. Statistical Analysis

The consumption of NOVA food groups and subgroups was assessed as dietary energy intake from ultra-processed foods (kcal) and the contribution of ultra-processed foods to total daily energy intake (%). The consumption of the NOVA food groups and subgroups of the total population (KNHANES from 2010 to 2018) was presented as means and standard errors.

We evaluated whether the dietary contribution of ultra-processed foods varied across sex, age, residence, education, and household income status using univariate and multiple regression analyses. In the multiple regression models, all of the covariates of the study were adjusted. Tests of linear trend were performed to assess the effect of sex, age, residence, education, and household income. We also evaluated whether the ultra-processed food consumption changed over time, as well as whether the time trends varied across socioeconomic strata, using linear regression analyses. The mean dietary contribution of ultra-processed foods across the three survey cycles (2010–2012, 2013–2015, and 2016–2018) were estimated in both the total population and each sociodemographic stratum. Tests of linear trend across the entire study period were performed. In addition, to help understand the change of ultra-processed food consumption by age groups, we estimated the dietary energy contribution of ultra-processed food subgroups over time by age groups. For this analysis, we regrouped a total of 12 ultra-processed food subgroups into six, as follows: Cereals, breads, cakes, and sandwiches; sugar-sweetened drinks (including coffee and tea with added sugar, sweetened milk and its products, and soft drinks, fruit and vegetable drinks); distilled alcoholic beverages; instant foods (including instant noodles and dumplings, fish and meat processed foods, instant cooked rice, soup, and other dishes); cookies, chips, snacks, and confectionary; and sauces and others (including traditional sauces and others). The mean dietary contribution of the ultra-processed food subgroups across the survey cycle was estimated and the adjusted linear trend was tested.

Given the clustered sampling design of KNHANES and the sample weights, we used the PROC SURVEYMEANS and PROC SURVEYREG procedures to explore the association between sociodemographic characteristics and the dietary contribution of ultra-processed foods, as well as the time trends in the ultra-processed food consumption, from 2010 to 2018. The data were analyzed using SAS 9.4 software (SAS institute, Cary, NC, USA). A *p*-value < 0.05 was considered statistically significant.

## 3. Results

### 3.1. Dietary Energy Intake according to the NOVA Food Groups

The mean energy intake of Koreans from 2010 to 2018 was 2024.9 kcal. Nearly two-thirds of the daily energy intake came from unprocessed or minimally processed foods (61.5%) and processed culinary ingredients (4.1%). Processed foods contributed 9.4% of the total energy intake, and the consumption of ultra-processed foods accounted for 24.9% (Table S1).

**Figure 1:**
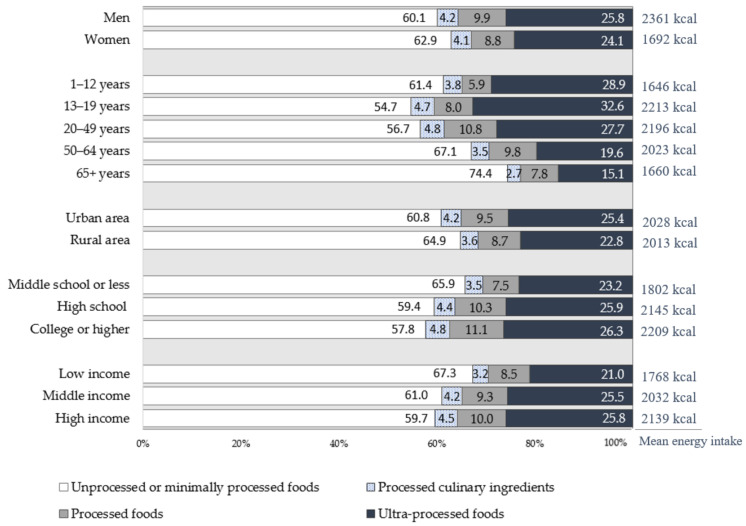
Dietary energy intake and energy contribution of the NOVA food groups by sociodemographic characteristics (KNHANES (Korea National Health and Nutrition Examination Survey) from 2010 to 2018).

The dietary energy intake and energy contribution of the four NOVA food groups according to socioeconomic characteristics is shown in Figure 1. In all socioeconomic subgroups, more than half of the total energy intake came from unprocessed or minimally processed foods, followed by ultra-processed foods, processed foods, and processed culinary foods. Although there were differences in degrees, such a tendency was found consistently in all groups.

### 3.2. Association between Socioeconomic Characteristics and the Dietary Contribution of Ultra-Processed Foods

The associations between socioeconomic characteristics and the dietary contribution of ultra-processed foods are presented in Table 1. On the whole, the crude mean contribution of ultra-processed foods was high among men and urban residents, and increased with education and income level; additionally, it reached its peak in adolescents (32.6%, 734.9 kcal) and thereafter decreased with increasing age (15.1%, 262.2 kcal in elderly aged 65 years or older). The linear trends in all socioeconomic characteristics were statistically significant (*p* < 0.0001). After adjustment for these socioeconomic variables, the adjusted mean contribution of the ultra-processed foods was still significantly higher in men (*p* < 0.0001), urban residents (*p* = 0.004), older people (*p* < 0.0001), and people with higher education (*p* < 0.0001), but a statistical difference across household income level was not found (*p* = 0.174).

### 3.3. Time Trends in the Dietary Contribution of Ultra-Processed Foods

The changes in the consumption of ultra-processed foods over time (2010–2018) are presented in Table 2. The overall contribution of ultra-processed foods increased gradually from 23.1% in 2010–2012 to 25.5% in 2013–2015 and 26.1% in 2016–2018 (+1.52% per cycle, *p* < 0.0001). Although there were slight differences in the extent of the increase in the dietary contribution of ultra-processed foods according to socioeconomic subgroups, significantly increasing trends were found in all sociodemographic strata. By age groups, adults aged 20–49 years had the largest change over time from 24.8% (564.1 kcal, data not shown) in 2010–2012 to 29.8% (668.7 kcal, data not shown) in 2016–2018 (+2.47% per cycle, *p* < 0.0001) compared to other age groups. Although the change over time was the smallest (+0.82% per cycle, *p* = 0.036), even among those with low household income, the consumption of ultra-processed foods increased consistently from 20.3% in 2010–2012 to 22.0% in 2016–2018. These increasing trends remained unchanged after adjustment for the socioeconomic variables (data not shown).

**Figure 2:**
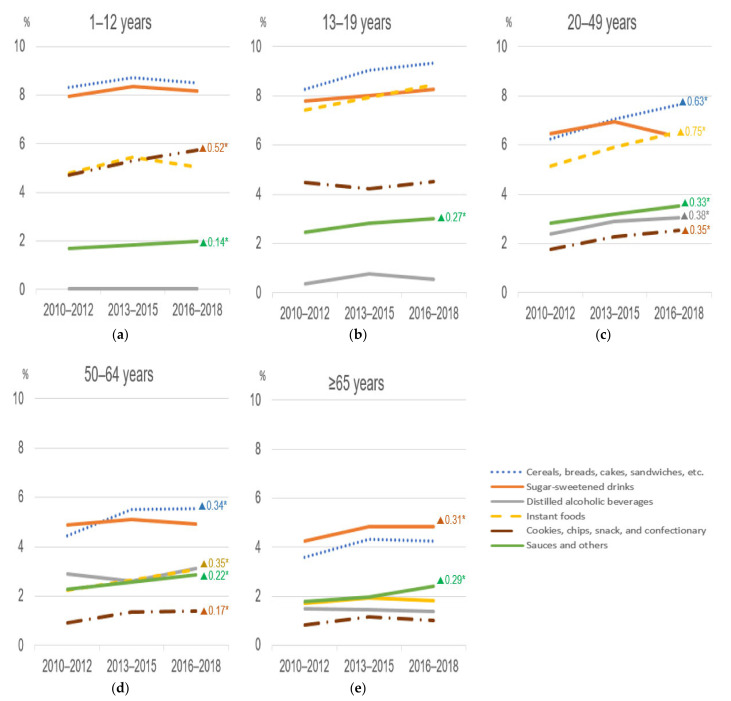
Time trends in the dietary energy contribution of ultra-processed foods’ subgroups by age group (KNHANES from 2010 to 2018. (**a**) Children aged 1–12 years, (**b**) adolescents aged 13–19 years, (**c**) adults aged 20–49 years, (**d**) adults aged 50–64 years, and (**e**) elders aged 65 years or older. “▲number*” means a statistically significant increasing change per cycle at *p* < 0.05 for adjusted linear trend tests. In the analyses, sex, residence area, education, and household income level were adjusted.

We also assessed the time changes in the dietary energy contribution of the subgroups within the ultra-processed foods group over time by age groups (Figure 2). In all age groups, a considerable portion of the dietary energy contribution of ultra-processed foods during the study period came from cereals, breads, cakes, sandwiches, and sugar-sweetened drinks, followed by instant foods. For children, a statistically significant increasing trend was found in the consumption of cookies, chips, snacks, and confectionary (+0.52% per cycle, *p* = 0.0003) and sauces and others (+0.14% per cycle, *p* = 0.001). For adolescents, the same significant trend was observed in the consumption of sauces and others (+0.27% per cycle, *p* = 0.001). Adults aged 20–49 years who showed the largest change during the study period over time had noticeably increasing trends in almost all ultra-processed food subgroups, ranging from +0.33% per cycle (in sauces and others, 2.8% in 2010–2012 to 3.5% in 2016–2018, *p* = 0.0001) to +0.75% per cycle (in instant foods, 5.1% in 2010–2012 to 6.6% in 2016–2018, *p* < 0.0001), except sugar-sweetened drinks. A similar linear trend (except sugar-sweetened drinks and distilled alcoholic beverages) was found for adults aged 50–64 years. Elderly people aged 65 years or older, although consuming less ultra-processed foods compared to the other age groups, showed a slight but significant increasing change in some subgroups, including sugar-sweetened drinks and sauces and others. For sugar-sweetened drinks, the consumption pattern differed slightly by age group (Table S2). Children and adolescents received more energy from sweetened milk and its products, soft drinks, and fruit and vegetable drinks, while adults and elderly people consumed more energy from coffee and tea with added sugar.

## 4. Discussion

This study investigated the association between socioeconomic characteristics and ultra-processed food consumption, and evaluated the change of the consumption of these foods in the past decade in Korea using the representative data from the KNHANES (2010–2018). The consumption of ultra-processed foods in Korea was higher among men, those living in urban areas, highly educated individuals, those with a high income, and younger individuals. Such associations remained significant even after adjustment for the other variables, but not in the case of income level. The contribution of ultra-processed foods to total energy intake gradually increased from 23.1% (2010–2012) to 26.1% (2016–2018), with +1.52% per cycle (*p* < 0.0001). The increasing trend over time was significantly found in all socioeconomic strata. A noticeable increasing trend was observed in adults aged 20–49 years, and foods with increased intake slightly differed by age groups.

In our study, men consumed more energy from ultra-processed foods than women, which is consistent with previous studies [3,10,25,42]. This seems to be because of differences between men and women in eating habits, nutritional behaviors, and health convictions [43]. However, in some studies conducted in Mexico [31], Chile [32], and Columbia [19], no significant difference in ultra-processed food consumption between sexes was found. Perhaps, the degree and pattern of sexual disparity in dietary consumption is likely to be different between high-income and low- or middle-income countries.

A notable negative association between age and ultra-processed food consumption has been consistently found in almost all previous studies [3,10,16,19,25,31,32,42,44,45]. Interestingly, the magnitude of the gap of ultra-processed food consumption between age groups seems to vary slightly by the country’s income level. In high-income countries such as the United States [16] and Canada [3], the energy contribution of the ultra-processed food consumption of children and adolescents was approximately one-third higher than older people (United States: 66.8% in adolescents and 52.8% in adults aged ≥60; Canada: 55.1% children and adolescents and 42.6% in adults aged ≥65 years), whereas the ultra-processed food consumption of children and adolescents of Chile [32] and Columbia [19] was almost twice as high as that of older people (Chile: 37.6% in children and adolescents and 17.4% in adults aged ≥65 years; Columbia: 18.5% in adolescents and 11.8% in adults aged ≥50 years). In our study, the dietary energy contribution of ultra-processed food was the highest in adolescents (32.6%, 734.9 kcal) and their consumption of ultra-processed food was two times more (in contribution of total energy intake, nearly three times in calories) than that of elderly people aged ≥65 years (15.1%, 262.2 kcal). Generally, younger people, including children and adolescents, are vulnerable to ultra-processed food consumption. There are several possible explanations for this [45,46]: Younger people prefer these foods and they pursue new food products and a new lifestyle. They also seem to respond more sensitively to food marketing and advertising. The relatively lower time, energy, and skill required for the preparation of meals may lead to the consumption of ultra-processed foods.

Socioeconomic status and residence area have also been shown to be significant factors associated with ultra-processed food consumption. According to previous studies, even the tendency of these associations varies by a country’s income level. In high-income countries [3,9,16,25], rural residents, less educated individuals, and those with low income consumed more ultra-processed foods than their counterparts. Contrary to this, in middle- and low-income countries [19,31,32,45,47,48], living in an urban area and high socioeconomic status (i.e., high education and high income) was strongly associated with higher consumption of ultra-processed foods. This can be attributed to differences in food price, affordability, and accessibility to those foods between countries [20,21,49]. Ultra-processed foods are relatively cheap in high-income countries, thus those foods seem appealing to poor individuals in those countries. However, in middle- or low-income countries, ultra-processed foods still cost more than natural foods, and the poor continue to choose to prepare their meals with relatively low-priced natural foods [49]. In addition, modern grocery retailers such as supermarkets, hypermarkets, and convenience stores, which have a considerable market share of ultra-processed foods, generally tend to spread from major cities to small localities, targeting wealthier individuals at first, and then poorer urban and rural residents [21]. In the rural areas of middle- or low-income countries, modern grocery retail channels are not prevalent yet, and thus rural residents may have less exposure to ultra-processed foods.

On the whole, Korea seems to exhibit unique characteristics regarding the consumption of ultra-processed foods and associations with socioeconomic status. Korea is a high income country, but the dietary energy contribution of ultra-processed foods is relatively low, close to that of middle- or low-income countries [3,10,16,17,18,19,31,32,45,50]. Our study showed that urban residents and highly educated individuals consume more ultra-processed foods. However, Korean society has been changing at a rapid speed (i.e., increases in single-person households and women working outside the home) [51]. Along with this, individual food preferences have changed toward the pursuit of taste and convenience, with household expenditures on fresh foods steadily decreasing and the convenience food industry expanding rapidly [52]. Moreover, Korea is also facing the considerable market power of transnational food and beverage corporations targeting Asian markets, which may result in a nutrition transition [20]. Similar to the findings observed in other countries [22,23,24,27], the current study found an increasing trend in the consumption of ultra-processed foods in Korea over the past decade (2010–2018), and this trend was significant across all socioeconomic strata [49].

Growing evidence has accumulated that the consumption of ultra-processed foods has positive associations with metabolic health [7,13,53,54,55,56,57,58,59] such as obesity, hypertension, dyslipidemia, and diabetes, cardiovascular diseases [12,14], cancer [60,61], and mortality [9,62,63]. The adverse impacts of ultra-processed foods on human health have been explained by the poor nutrient profile of these foods [3,17], the low dietary quality [3,4,11,33] associated with the consumption of these foods, and the addictive eating behaviors induced by these foods, which may lead to overconsumption [7,64]. The consumption of ultra-processed foods has also been found to have positive relationships with an unhealthy lifestyle [4,7,9,10,11]. Moreover, ultra-processed foods contain various types of food constituents that are created in the process of producing, processing, and packaging food: cosmetic additives (such as preservatives, emulsifiers, sweeteners, etc.); contaminants neoformed in processing (such as acrylamide and acrolein); trace chemicals from food packaging materials (such as Bisphenol A and phthalates) [65,66,67,68]. Recently, evidence has emerged that these food constituents can reduce the diversity of the gut microbiota, alter the interactions of the host microbiota, and thus contribute to the development of metabolic syndrome and inflammatory diseases [65,66,69] which require consistent attention.

To the best of our knowledge, this is the first study to evaluate the time trends in the ultra-processed food consumption of Koreans and to investigate the association with socioeconomic characteristics. We used the data from KNHANES, which is a nationally representative survey. This survey is conducted throughout the year and the diet recall days are distributed from Monday to Sunday; thus, unbiased statistics of a specific season or day can be obtained. Generally, the food purchase records collected from a household’s food expenditure survey do not include food consumed outside the home or food wastage in the home, and therefore cannot reflect accurate dietary intake [22,23,55]. The dietary data we analyzed were the most up-to-date, as the dietary consumption data were collected at the individual level [37]. Thus, we were able to evaluate the findings on the consumption of each individual. Raw dietary data included information on the composition of ingredients of dishes and the amount consumed per each ingredients [41]. Thus, we were able to use the disaggregated food information of freshly prepared meals. In addition, the NOVA food classification system we utilized for the identification of ultra-processed foods is widely used for classifying foods based on the nature, extent, and purpose of industrial food processing [1,70].

Despite these strengths, there are limitations that should be considered. First, the dietary data used in the study were assessed by a one-day 24 h recall, which was not able to reflect the usual intake of the population. However, one-day recall is sufficiently acceptable when used for estimating the average intake of a population, rather than for individuals’ usual intake [71]. Second, although dietary assessment was performed according to a predefined standardized protocol [37,39], 24 h recalls are subjective to measurement errors, especially with distorted self-reports of dietary intake [72]. On the whole, unhealthy foods are more likely to be underreported due to social desirability bias [73], leading to an underestimation of ultra-processed food consumption. Third, KNHANES has collected a considerable amount of information (such as information for dishes prepared at or outside the home, detailed composition of ingredients, and more specific descriptions of each ingredient), which the Food and Agriculture Organization of the United Nations provides guidance for in terms of collecting information on food processing through food consumption surveys [74]. However, KNHANES was not designed to assess food consumption classified by degree of food processing, and thus some of the descriptions were not sufficient to categorize foods according to the NOVA system. Moreover, some convenient foods (i.e., ready-to-eat soybean paste stew) could have been classified as dishes (prepared at home or outside the home), likely underestimating consumption of ultra-processed foods in Koreans.

## 5. Conclusions

This study showed that ultra-processed food consumption in Korea in the period of 2010–2018 was higher among men, younger individuals, urban residents, and more educated people, and gradually increased during the last decade. Although the contribution of ultra-processed food consumption varies by socioeconomic characteristics, an increasing trend was consistently found in all sociodemographic strata. Food consumption is influenced by food supply chains, food environments, policy and regulatory frameworks, in addition to individuals’ behavior [21]. Our results suggest that strategies to decrease the consumption of ultra-processed foods and to promote a healthy diet among the Korean population should be sought. Given the consistently increasing consumption of ultra-processed foods in all socioeconomic groups, strategies for the entire population are required. Furthermore, given the differences across socioeconomic subgroups, different approaches for vulnerable groups are necessary. As such, further studies are needed to identify the drivers that induce the purchase and consumption of ultra-processed food, and to help us understand the barriers preventing us from controlling the consumption of these foods.

## Figures and Tables

**Table 1:** Dietary energy contribution of ultra-processed foods according to sociodemographic characteristics (KNHANES from 2010 to 2018).

	Dietary Contribution of Ultra-Processed Foods to Total Energy Intake (%)			
Variables	Crude Mean (95% CI)		Adjusted Mean (95% CI) ^1^	
Sex				
Male	25.7	(25.5–26.1)	25.8	(25.5–26.1)
Female	24.1	(23.8–24.4) *	25.0	(24.4–25.6) *
Age (years)				
1–12	28.9	(28.5–29.4)	30.7	(30.0–31.3)
13–19	32.6	(31.9–33.4)	33.8	(32.9–34.6)
20–49	27.7	(27.3–28.0)	26.6	(26.1–27.0)
50–64	19.6	(19.2–19.9)	19.7	(19.3–20.1)
≥65	15.1	(14.8–15.8) *	16.3	(15.8–16.7) *
Residence				
Urban	25.4	(25.1–25.6)	25.8	(25.5–26.1)
Rural	22.8	(22.1–23.4) *	25.0	(24.4–25.6) *
Education				
Middle school or less	23.2	(22.8–23.5)	23.4	(23.0–23.8)
High school	25.9	(25.5–26.3)	26.4	(25.9–26.9)
College or higher	26.3	(26.0–26.7) *	26.3	(25.8–26.9) *
Household income				
Low (Q1)	21.0	(20.4–21.6)	25.5	(24.9–26.1)
Middle (Q2–Q3)	25.5	(25.2–25.8)	25.4	(25.0–25.8)
High (Q4)	25.8	(25.4–26.2) *	25.3	(24.8–25.7)

Korean population aged one year or older (KNHANES from 2010 to 2018). ^1^ Adjusted for all of the other variables presented in the table. * *p* for linear trend <0.05.

**Table 2:** Time trends in the dietary contribution of ultra-processed foods according to sociodemographic characteristics (KNHANES from 2010 and 2018).

	Dietary Contribution of Ultra-Processed Foods to Total Energy Intake (%)			Changes per Cycle	*p* for Trend
Variables	2010–2012	2013–2015	2016–2018		
Total	23.1 (22.7–23.5)	25.5 (25.1–25.9)	26.1 (25.7–26.5)	1.52	<0.0001
Sex					
Male	23.7 (23.2–24.2)	26.5 (25.9–27.0)	27.1 (26.6–27.6)	1.72	<0.0001
Female	22.5 (22.0–23.0)	24.6 (24.2–25.1)	25.2 (24.6–25.7)	1.32	<0.0001
Age (years)					
1–12	27.5 (26.7–28.3)	29.8 (29.9–30.6)	29.6 (28.9–30.4)	1.06	0.0002
13–19	30.9 (29.7–32.1)	32.9 (31.7–34.2)	34.4 (33.1–35.6)	1.74	0.0001
20–49	24.8 (24.3–25.4)	28.4 (27.8–29.0)	29.8 (29.2–30.4)	2.47	<0.0001
50–64	17.6 (17.0–18.2)	19.8 (19.2–20.4)	20.9 (20.3–21.5)	1.64	<0.0001
≥65	13.7 (13.1–14.3)	15.7 (15.1–16.2)	15.8 (15.2–16.4)	0.98	<0.0001
Residence					
Urban	23.7 (23.2–24.1)	25.8 (25.4–26.3)	26.5 (26.1–26.9)	1.40	<0.0001
Rural	20.7 (19.6–21.7)	24.1 (23.1–25.2)	24.0 (22.8–25.2)	1.72	<0.0001
Education					
Middle school or less	21.7 (21.2–22.2)	24.0 (23.4–24.6)	24.0 (23.4–24.6)	1.18	<0.0001
High school	24.0 (23.3–24.7)	26.5 (25.8–27.3)	27.3 (26.6–28.0)	1.67	<0.0001
College or higher	24.4 (23.8–25.1)	26.6 (26.0–27.3)	27.4 (26.8–28.0)	1.45	<0.0001
Household income					
Low (Q1)	20.3 (19.2–21.4)	20.7 (19.8–21.6)	22.0 (20.9–23.1)	0.82	0.0361
Middle (Q2–Q3)	23.4 (22.9–23.9)	26.5 (26.0–27.1)	26.7 (26.1–27.2)	1.62	<0.0001
High (Q4)	23.9 (23.3–24.6)	26.0 (25.3–26.6)	27.2 (26.5–27.8)	1.60	<0.0001

Korean population aged one year or older (KNHANES from 2010 to 2018).

## Data Availability

Data were obtained from the Korea National Health and Nutrition Examination Survey (KNHANES) and are available online at the KNHANES website (https://knhanes.cdc.go.kr, accessed on 20 February 2020).

## Supplementary Materials

The following are available online at https://www.mdpi.com/article/10.3390/nu13041120/s1, Table S1, Dietary energy intake according to the NOVA food groups (Korea National Health and Nutrition Examination Survey (KNHANES) from 2010 to 2018), Table S2: Consumption of ultra-processed foods and subgroups by age groups (KNHANES from 2010 to 2018).
