# Supplementary material for: Socioeconomic Characteristics and Trends in the Consumption of Ultra-Processed Foods in Korea from 2010 to 2018
Source: Nutrients. 2021 Mar 29;13(4):1120. doi: 10.3390/nu13041120 (PMC8065678; doi:10.3390/nu13041120)
Supplement: Supplementary file 1 [file nutrients-13-01120-s001.zip › Supplementary materials/Table S1. Dietary energy intake according to the NOVA food groups.docx]

**Table S1.** Dietary energy intake according to the NOVA food groups (Korea National Health and Nutrition Examination Survey (KNHANES) from 2010 to 2018).

|  |  | **Energy Intake**  **(kcal)** | |  | **Contribution to Total Energy Intake (%)** | |
| --- | --- | --- | --- | --- | --- | --- |
| **Food Group** |  | **Mean** | **(SE)** |  | **Mean** | **(SE)** |
| **Unprocessed or minimally processed foods** |  | **1203.9** | **(4.1)** |  | **61.5** | **(0.1)** |
| Grains |  | 683.4 | (2.8) |  | 36.3 | (0.1) |
| Meats |  | 182.1 | (1.9) |  | 7.9 | (0.1) |
| Fruits |  | 80.7 | (1.0) |  | 4.3 | (0) |
| Vegetables, mushrooms, and seaweeds |  | 62.6 | (0.4) |  | 3.1 | (0) |
| Fish and sea foods |  | 43.0 | (0.6) |  | 2.0 | (0) |
| Milk and plain yogurts |  | 42.2 | (0.5) |  | 2.4 | (0) |
| Eggs |  | 40.9 | (0.4) |  | 2.1 | (0) |
| Legumes, nuts, and seeds |  | 34.6 | (0.4) |  | 1.8 | (0) |
| Potatoes |  | 29.9 | (0.7) |  | 1.5 | (0) |
| Coffee and tea without sugar |  | 4.5 | (0.1) |  | 0.2 | (0) |
| **Processed culinary ingredients** |  | **88.8** | **(0.7)** |  | **4.1** | **(0)** |
| Plant oils |  | 58.8 | (0.6) |  | 2.7 | (0) |
| Sugars (sugar, honey, molasses, etc.) |  | 23.1 | (0.3) |  | 1.1 | (0) |
| Animal fats |  | 3.4 | (0.2) |  | 0.2 | (0) |
| Starch |  | 3.3 | (0.1) |  | 0.1 | (0) |
| Salt, vinegars, and others |  | 0.2 | (0) |  | 0.0 | (0) |
| **Processed foods** |  | **200.6** | **(2.0)** |  | **9.4** | **(0.1)** |
| Noodles and starchy jello |  | 96.2 | (1.5) |  | 4.5 | (0.1) |
| Fermented alcoholic beverages |  | 34.8 | (0.8) |  | 1.3 | (0) |
| Salted or pickled vegetables, including Kimchi |  | 31.1 | (0.2) |  | 1.6 | (0) |
| Soybean curd |  | 17.9 | (0.3) |  | 0.9 | (0) |
| Canned or bottled fish and sea foods |  | 11.7 | (0.4) |  | 0.6 | (0) |
| Fruit jams and canned fruits |  | 4.5 | (0.1) |  | 0.2 | (0) |
| Others (seasoned nuts, seeds, cheese, etc.) |  | 6.2 | (0.3) |  | 0.3 | (0) |
| **Ultra-processed foods** |  | **531.4** | **(3.3)** |  | **24.9** | **(0.1)** |
| Cereals, breads, cakes, sandwiches, etc. |  | 139.4 | (1.7) |  | 6.6 | (0.1) |
| Distilled alcoholic beverages |  | 62.3 | (1.5) |  | 2.1 | (0) |
| Coffee and tea with added sugar ^1^ |  | 50.9 | (0.5) |  | 2.7 | (0) |
| Instant noodles and dumplings |  | 48.4 | (1.1) |  | 2.4 | (0) |
| Sweetened milk and its products |  | 39.2 | (0.7) |  | 2.1 | (0) |
| Fish and meat processed foods |  | 40.5 | (0.8) |  | 1.9 | (0) |
| Cookies, chips, and snacks |  | 36.5 | (0.8) |  | 1.8 | (0) |
| Soft drinks and fruit and vegetable drinks |  | 32.5 | (0.6) |  | 1.5 | (0) |
| Traditional sauces |  | 27.5 | (0.2) |  | 1.3 | (0) |
| Confectionary |  | 12.6 | (0.4) |  | 0.6 | (0) |
| Instant cooked rice, soup, and other dishes |  | 9.0 | (0.3) |  | 0.4 | (0) |
| Others (instant sauces, condiment, etc.) |  | 31.1 | (0.5) |  | 1.4 | (0) |
| **All foods** |  | **2024.9** |  |  | **100** |  |

Korean population aged one year or older (KNHANES from 2010 to 2018).

^1^ Includes coffee or tea products with added sugar or milk, cocoa, or other sugar-sweetened beverages.
