# Supplementary material for: Socioeconomic Characteristics and Trends in the Consumption of Ultra-Processed Foods in Korea from 2010 to 2018
Source: Nutrients. 2021 Mar 29;13(4):1120. doi: 10.3390/nu13041120 (PMC8065678; doi:10.3390/nu13041120)
Supplement: Supplementary file 1 [file nutrients-13-01120-s001.zip › Supplementary materials/Table S2. Consumption of ultra-processed foods and subgroups by age groups (KNHANES from 2010 to 2018).docx]

Table S2. Consumption of ultra-processed foods and subgroups by age groups (KNHANES from 2010 to 2018).

|  | Age group | | | | |
| --- | --- | --- | --- | --- | --- |
| Food groups | 1–12 years | 13–19 years | 20–49 years | 50–64 years | 65+  years |
| Ultra-processed foods (kcal) | 492.4 | 734.7 | 631.6 | 420.9 | 262.2 |
| Subgroups of ultra-processed foods (%) ^1^ |  |  |  |  |  |
| Cereals, breads, cakes, sandwiches, etc. | 27.7 (0.4) | 23.4 (0.5) | 20.6 (0.2) | 19.3 (0.3) | 15.8 (0.4) |
| Coffee and tea with added sugar | 3.0 (0.1) | 4.6 (0.2) | 14.4 (0.2) | 20.4 (0.3) | 25.2 (0.4) |
| Sweetened milk and its products | 17.2 (0.4) | 12.2 (0.4) | 6.2 (0.1) | 5.8 (0.2) | 6.0 (0.2) |
| Soft drinks and fruit and vegetable drinks | 6.9 (0.2) | 9.5 (0.3) | 6.5 (0.1) | 3.8 (0.1) | 3.0 (0.1) |
| Distilled alcoholic beverages | 0.1 (0.0) | 1.3 (0.2) | 7.5 (0.2) | 9.1 (0.3) | 5.2 (0.2) |
| Fish and meat processed foods | 9.3 (0.2) | 10.3 (0.4) | 8.8 (0.2) | 4.9 (0.2) | 2.8 (0.1) |
| Instant noodles and dumplings | 5.5 (0.2) | 9.6 (0.4) | 7.5 (0.2) | 4.3 (0.2) | 3.5 (0.2) |
| Instant cooked rice, soup, and other dishes | 1.4 (0.1) | 1.8 (0.1) | 1.6 (0.1) | 0.8 (0.1) | 0.7 (0.1) |
| Cookies, chips, and snacks | 12.3 (0.3) | 9.5 (0.4) | 5.6 (0.1) | 3.9 (0.2) | 3.3 (0.2) |
| Confectionary | 5.2 (0.2) | 3.7 (0.2) | 1.8 (0.1) | 1.7 (0.1) | 2.9 (0.1) |
| Traditional sauces | 5.2 (0.2) | 6.5 (0.3) | 10.7 (0.2) | 19.1 (0.3) | 25.5 (0.4) |
| Others (instant sauces, condiments, etc.) | 5.5 (0.2) | 7.5 (0.3) | 8.0 (0.1) | 6.0 (0.2) | 4.7 (0.2) |

^1^ Proportion (%) of each subgroup consumption (kcal) to total consumption of ultra-processed foods (kcal). Data are presented as mean (SE).
